# Primary bronchial Ewing sarcoma^⋆^

**DOI:** 10.1016/j.ijscr.2019.07.062

**Published:** 2019-07-25

**Authors:** Hamid Mumtaz, Farah Khalil, Amit Tandon, Eric Toloza, Jacques-Pierre Fontaine

**Affiliations:** Moffitt Cancer Center, 12902 USF Magnolia Drive, Tampa, FL, 33612, USA

**Keywords:** Primary bronchial Ewing sarcoma

## Abstract

**Introduction:**

Primary bronchial Ewing sarcoma (ES) is a rare endobronchial tumor.

**Presentation of case:**

A 65-year-old male presented with six-month history of progressive shortness of breath. Flexible bronchoscopy showed an endobronchial polypoid tumor in the left main stem bronchus about 2 cm from the carina. The tumor was resected by a left bronchial sleeve resection using a right postero-lateral thoracotomy approach. Pathology showed complete tumor resection with negative margins. The morphological and immune-phenotypical features of the resected specimen were compatible with ES. He had an uneventful post-operative recovery. He did not receive adjuvant radiation or chemotherapy and remains disease free at 9 months follow up.

**Discussion:**

A review of the literature identified six other cases of primary bronchial ES. In addition, there were three reported cases of primary ES involving the trachea and thirteen involving the lung parenchyma. Bronchial ES appeared to have a relatively better prognosis than ES involving the trachea or the lung. Our case demonstrates that primary bronchial ES may be treated safely with limited resection, lung preservation and without the need for adjuvant therapy if negative margins can be achieved.

**Conclusion:**

Sleeve resection without adjuvant therapy may be a safe treatment option for primary bronchial ES.

## Highlights

• Primary Ewing sarcoma of the respiratory system (trachea, bronchus and the pulmonary parenchyma) are rare tumors.
• Bronchial Ewing sarcoma may be managed safely with sleeve resection and lung preservation.
• Adjuvant therapy may not be needed if the tumor can be resected safely with negative margins.

## 1 Introduction

Ewing sarcoma/peripheral neuroectodermal tumors are a member of the Ewing sarcoma family of tumors (ESFT). They arise from undifferentiated neuroectodermal cells and share a common neural histogenesis and tumor genetics [1]. They typically involve the long and flat bones (skeletal ESFT) of children and adolescents but have been reported in multiple other tissue sites (extra-skeletal ESFT) in both children and older patients. Primary ES tumors of the respiratory system (trachea, bronchus and the pulmonary parenchyma) are however rare, and only handful of cases have been reported thus far in the literature with different treatment strategies [2,3].We report our experience in the management of this rare tumor.

This case report has been written in line with the SCARE guidelines [4].

## 2 Presentation of case

A 65-year-old white male presented with symptoms of progressive shortness of breath and cough over a 6-month period. His past medical history was significant for a minor stroke at age 43 years due to a drug overdose with residual concentration and memory deficit. Otherwise, he had well-controlled hypertension, dyslipidemia, benign prostatic hypertrophy, and depression. He had an uneventful left hip replacement about 5 months prior to presentation. He had a 20-pack year history of smoking cigarettes and had quit a year ago. His family history included his father who had bladder cancer and mother with coronary artery disease. His physical examination was within normal limits.

Computed tomographic (CT) of chest with intravenous contrast demonstrated numerous punctate lung nodules measuring less than 3 mm as well as a 16 mm partially occluding endobronchial mass in the left main stem bronchus. Flexible bronchoscopy showed a polypoid mass in the left main stem bronchus. Initial biopsy was suggestive of a sarcoma. Positron emission tomography (PET) scan showed mild FDG avid endobronchial lesion (SUV 3.2), hypermetabolic consolidative process in the lingula (SUV 7.3) and left lower lobe (SUV 8.2). The micronodules seen on CT were below the resolution of PET scan. A bone scan and Brain MRI scan showed no signs of metastatic disease.

The patient was referred to our thoracic surgery clinic after the above work up. We repeated the bronchoscopy that included both a flexible and rigid scope. The left main stem tumor appeared as a polypoid, whitish, firm lesion about 2 cm from the main carina. The lesion was causing a near complete obstruction of the left main stem with a ball-valve effect. A partial endoscopic resection of the tumor was performed to improve ventilation to the left lung. Patient’s dyspnea and cough markedly improved after the procedure.

**Fig. 1:**
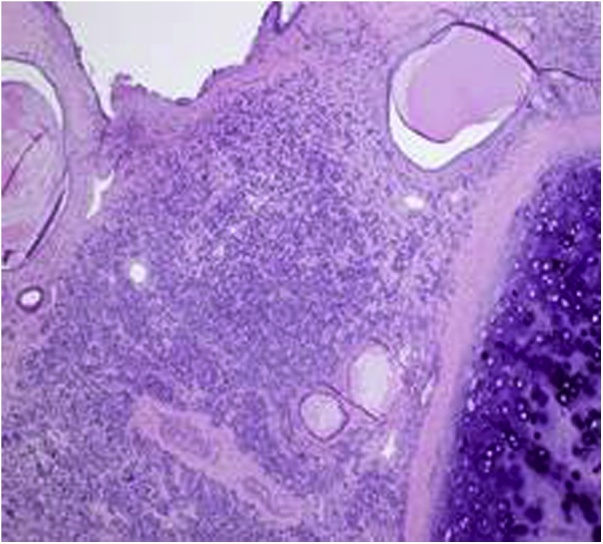
Bronchial Ewing sarcoma with a dense cellular infiltration involving the bronchial wall (4x magnification).

**Fig. 2:**
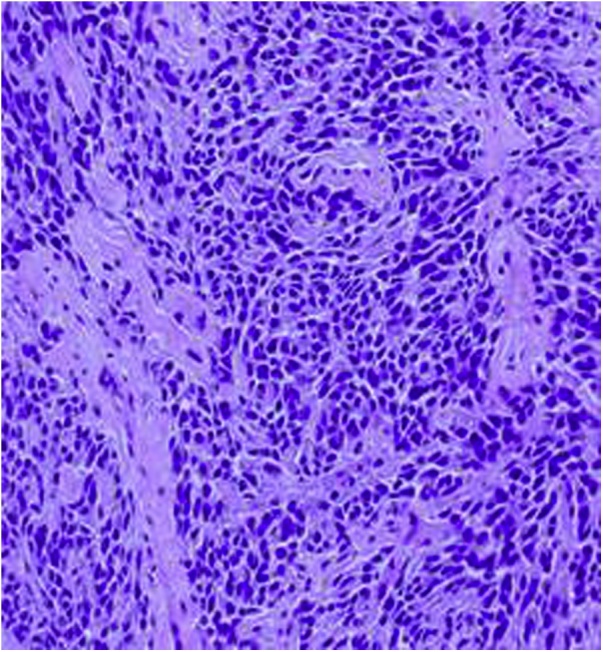
Bronchial Ewing sarcoma demonstrating invasive sheets of small round blue cells with indistinct cell membranes (20× magnification).

Pathology showed the tumor was composed of uniform small round cells in a solid architecture (Figs. 1 & 2 ). The mitosis rate was 2 per 10 high power field with no necrosis. Immuno-stains with adequate controls showed the tumor cells to be positive for CD99 and bcl2, but negative for CD56, TTF1, S100 and CD57. Manual morphometric analysis for Ki-67 showed 15% of tumor cells to be positive. The morphologic and immune-phenotypical findings were compatible with Ewing sarcoma (ES). FISH studies were negative for rearrangement of the ESWR1 (22q12) locus in addition to negative SS18/SSX1 and SS18/SSX2 fusion transcript for synovial sarcoma, suggesting this tumor may be rare variant of ES family of tumors.

**Fig. 3:**
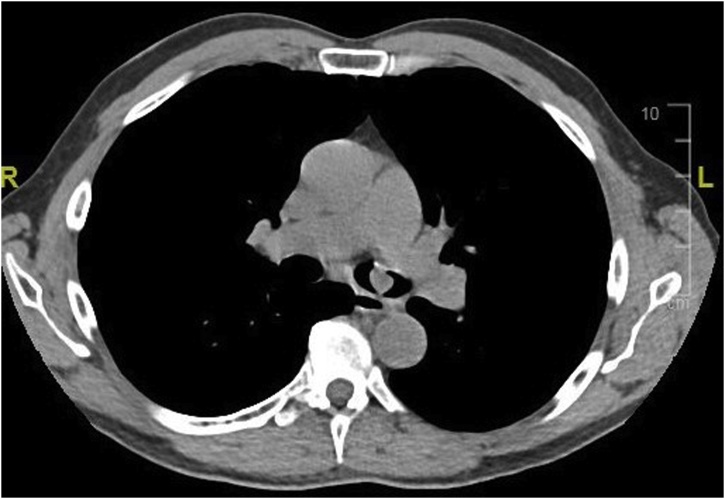
CT chest shows a left endobronchial tumor.

A subsequent CT chest with intravenous contrast showed expected interval decrease in size of the left endobronchial tumor as well as complete resolution of the tiny peripheral nodules and the left lung consolidative process suggesting an inflammatory process due to prior airway obstruction (Fig. 3).

Patient was presented at our Multidisciplinary Sarcoma Tumor Board. The recommendation was to proceed with surgical resection as this was the only site of disease. Our surgical plan was to proceed with left bronchial sleeve resection with primary anastomosis through a right posterolateral thoracotomy due to proximity of the tumor to the main carina. The chest was entered through the fifth intercostal space including harvesting the intercostal muscle as a long pedicled muscle flap. The subcarinal level 7 nodal station was removed allowing excellent exposure of the left main stem bronchus and the main carina. Intra-operative flexible bronchoscopy was used to confirm the location of the tumor and guide in resection with negative margins. The two ends of the left main stem bronchus were then re-anastomosed without tension using interrupted 2-0 Vicryl sutures. The intercostal muscle flap was used to buttress the bronchial suture line. Patient was extubated without any difficulty. Post-operative chest roentgenogram showed well expanded lungs with no pneumothorax. There was no air leak noted. He did have a low volume mild chylous pleural drainage for the first two post-operative days that resolved with low fat dietary management. The chest tubes were removed on the fifth post-operative day and he was discharged home on day 6.

Gross examination of the resected specimen showed an exophytic sessile rubbery tumor arising from the bronchial wall with adequate resection margins. The morphological and immune-phenotypical features were like the previously resected specimen.

Clinical and imaging follow up using CT chest with intravenous contrast at 3 months intervals showed no evidence of recurrence. At 9-month post-operative follow up, patient was doing well and Alpine skiing. His case was presented once again at the Tumor Board. Seeing that the tumor was completely resected with negative margins, and no signs of metastatic disease, the recommendation was to keep the patient under strict surveillance without adjuvant chemo-radiation therapy

Written informed consent was obtained from the patient for publication of this case report and accompanying images. A copy of the written consent is available for review by the Editor-in-Chief of this journal on request.

## 3 Discussion

The diagnosis of ES depends on histopathologic and immunohistochemical findings. Histological examination shows the tumor to be composed of diffuse compact sheet of small round cells that strongly express MIC-2 gene product (CD99). Although the later expression is not in unique to ES, over 95% of them are CD99 positive. Nonrandom chromosomal translocation (22q12) involving the Ewing sarcoma breakpoint region gene 1 are reported to be present in ESFT but have also been observed in clear cell sarcomas and desmoplastic small round cell tumors. Fluorescence in situ hybridization (FISH) has a high specificity 100% but only a moderate sensitivity 50–60% in the diagnosis of ES [5]

Our patient represents the seventh reported case of primary bronchial ES in the literature (Table 1). It is the first reported case of primary bronchial ES treated safely by a sleeve resection with lung preservation and without adjuvant therapy. The treatment of the other patients reported in the literature is detailed in Table 1. In the 7–18 months follow-up period, no patients had been reported dead in the series. Three case reports of primary tracheal ES have also been reported in the literature; one was treated by neoadjuvant chemotherapy followed carinal sparing tracheal resection with adjuvant chemoradiation therapy [6], and two were treated with bronchoscopic resection [7,8], only one received adjuvant radiation therapy [8]. All three patients were alive with no evidence of recurrence at 3–14 months follow up.

**Table 1:** Case reports of primary bronchial Ewing Sarcoma published in the literature.

Author	Age/gender	Location	Size(mm)	Surgery	Treatment	Follow up
[12]	18 /M	Right middle lobe bronchus	40	Lobectomy	None	24 months, local recurrence
[13]	22/F	Bronchus intermedius	50	None	Neoadjuvant chemotherapy and adjuvant chemoradiation	Not available
[3]	12/M	Right main bronchus	18	Bronchoscopic resection	Adjuvant chemoradiation	12 months, no recurrence
[14]	12/M	Right main bronchus	20	Bronchoscopic resection	Adjuvant chemoradiation	18 months, no recurrence
[9]	29/M	Left main bronchus	40	Left pneumonectomy	None	18 months, no recurrence
[2]	30/M	Left lower lobe bronchus	60	Left lower lobectomy	Adjuvant chemotherapy	18 months, no recurrence
Our case	64/M	Left main bronchus	16	Sleeve bronchial resection	None	9 months, no recurrence

Bronchial ES tumors are usually pedunculated, exophytic tumors involving a portion of the bronchial circumference and may be amenable to snare resection through a bronchoscope, however this approach may be associated with a higher risk of local recurrence as these tumors may invade into the underlying submucosa, muscle and cartilage [9]. While tumors in the distal bronchial airway and pulmonary parenchyma may need a lobectomy, tumors in the proximal trachea bronchial airway can be treated safely with sleeve resection when possible as illustrated in our patient.

Surgical resection with negative margins when possible appears to offer the best chance for disease free survival compared with additional use of neoadjuvant or adjuvant therapy. In one series of patients with localized extra-skeletal ES who underwent wide local excision, overall 5-year survival was 100%, patients who underwent suboptimal resection had an overall 5 year-survival of 58%. No other variables such as tumor size, location, stage of disease or radiation therapy were found to improve survival [10]. A case of cardiac ESFT has also been treated successfully with surgery only and without adjuvant chemotherapy, and the patient was well at 2 year-follow up [11].

We agree with the observations made by Chen and colleagues that the clinical features, curative effects and prognosis of primary trachea-bronchial ES may be different and probably better than those of pulmonary ESFT [2]. In a literature review of primary pulmonary ESFT, of the thirteen cases identified, five had died within a 5–24 months follow up period [3].

Based on the current evidence in the literature, our group’s recommendation for the treatment of trachea bronchial ESFT would be to perform up front a complete surgical resection while attempting to preserve as much lung parenchyma by using sleeve resection techniques. Neoadjuvant systemic therapy would be recommended if lesions are not amenable to complete surgical resection at the time of presentation and reserve adjuvant chemoradiation for patients with positive margins.

## Sources of funding

None.

## Ethical approval

Study is exempt from ethical approval as patient received standard treatment.

## Consent

Written informed consent was obtained from the patient for publication of this case report and accompanying images. A copy of the written consent is available for review by the Editor-in-Chief of this journal on request.

## Author contribution

All authors were involved in the management of this patient and in preparation of the manuscript.

Dr Farah Khalil: pathology interpretation.

Dr Amit Tandon- interventional pulmonary medicine – assisted with rigid/flexible bronchoscopy.

Dr Eric Toloza, Dr Jacques Fontaine, Dr Hamid Mumtaz: performed the surgery, and peri-operative care of the patient.

## Registration of research studies

Not applicable.

## Guarantor

Dr Jacques-Pierre Fontaine.

## Provenance and peer review

Not commissioned, externally peer-reviewed.

## Declaration of Competing Interest

No conflict of interest for all authors
